# The ceramide [NP]/[NS] ratio in the stratum corneum is a potential marker for skin properties and epidermal differentiation

**DOI:** 10.1186/s12895-020-00102-1

**Published:** 2020-08-31

**Authors:** Urara Yokose, Junko Ishikawa, Yuki Morokuma, Ayano Naoe, Yosuke Inoue, Yuka Yasuda, Hisashi Tsujimura, Tsutomu Fujimura, Takatoshi Murase, Atsushi Hatamochi

**Affiliations:** 1 Biological Science Research, Kao Corporation, 2606 Akabane, Ichikai-machi, Haga-gun, Tochigi, 321-3497 Japan; 2 Health and Beauty Research, Kao Corporation, 2-1-3 Bunka, Sumida-ku, Tokyo, 131-8501 Japan; 3 Analytical Science Research, Kao Corporation, 2606 Akabane, Ichikai-machi, Haga-gun, Tochigi, 321-3497 Japan; 4 Department of Dermatology, Dokkyo Medical University, 880 Kitakobayashi, Mibu-machi, Shimotsuga-gun, Tochigi, 321-0293 Japan

**Keywords:** Ceramide, Cer [NP]/[NS] ratio, Stratum corneum, Keratinocytes, Atopic dermatitis, Psoriasis

## Abstract

**Background:**

Specific species of ceramides (Cer), major constituents of lipids in the stratum corneum (SC), are decreased and are correlated with SC barrier and water-holding functions in the skin of patients with atopic dermatitis (AD) or psoriasis (Pso). However, possible correlations between Cer subclass ratios and skin properties in barrier-disrupted skin and in healthy skin remain unclear. The objective of this study was to identify a new marker to evaluate skin properties and epidermal differentiation in SC not only in barrier-disrupted skin but also in healthy skin.

**Methods:**

The Cer subclass ratios in the SC of healthy control subjects and in patients with AD or Pso were evaluated. Correlations with candidate markers and facial skin features of healthy Japanese females (20–74 years old, *n* = 210) were investigated. Variations of markers during epidermal differentiation were studied in human epidermis and in cultured keratinocytes.

**Results:**

The ratios of Cer [NP]/[NS], Cer [NH]/[NS], Cer [NP]/[AS], Cer [NH]/[NS], Cer [NDS]/[AS], Cer [AH]/[AS] and Cer [EOP]/[AS] showed significant differences between non-lesional skin of AD patients and normal skin of healthy control subjects, as well as Pso patients and their healthy control subjects. The Cer [NP]/[NS] ratio was correlated with SC functional parameters (transepidermal water loss and capacitance) and with skin appearance (texture, scaling and color) even in the cheek skin of healthy female subjects. The Cer [NP]/[NS] ratio in the SC was approximately 18-times higher than in living keratinocytes, and it increased as they differentiated.

**Conclusions:**

The Cer [NP]/[NS] ratio in the SC is a potential marker for skin properties and epidermal differentiation in barrier-disrupted skin as well as in healthy skin.

## Background

The stratum corneum (SC) is the outermost layer of the epidermis and plays a role as a critical permeability barrier [1]. The SC is composed of terminally differentiated keratinocytes (KCs) and extracellular lipids, predominantly ceramides (Cer), cholesterol and free fatty acids that form lipid lamellae [2]. Cer represent the major lipid class by mass (~ 50%) and have diverse molecular structures that are classified into 12 subclasses in human SC [3, 4]. The typical Cer subclasses detected in the SC are comprised of non-OH fatty acids [N], α-OH fatty acids [A], esterified ω-OH fatty acids [EO], dihydrosphingosines [DS], sphingosines [S], 6-hydroxy sphingosines [H] and phytosphingosines [P] [3–5]. In addition to those subclasses, there is a further subdivision according to the carbon chain length, resulting in approximately 480 types of Cer reported to exist in human SC [6, 7].

Differences in the profiles of SC lipids in skin from patients with atopic dermatitis (AD) and/or psoriasis (Pso) have been demonstrated [5, 8, 9]. Regarding Cer subclasses, reduced levels of Cer [EOS] and Cer [NP] in patients with AD or Pso have been proposed to be important to the SC barrier abnormality [10, 11]. We also reported the characteristic differences of Cer profiles in the SC of patients with AD or Pso represented by significant decreases in the levels of Cer [NH] and Cer [NP] compared with healthy control subjects [8, 9]. Further, Cer [NH] and Cer [NP] showed a significant negative correlation with transepidermal water loss (TEWL) and a positive correlation with capacitance, while Cer [NS] and Cer [AS] showed a tendency for a positive correlation with TEWL and a negative correlation with capacitance [8]. On the other hand, no Cer subclass in the SC showed a significant difference between the normal skin of healthy control subjects and the non-lesional skin of patients with AD or Pso [12].

In addition to Cer subclass differences, alterations in the composition of SC lipids represented by decreasing the Cer/cholesterol ratio or the lipid/protein ratio in AD skin compared to normal skin has been demonstrated to be related to the skin barrier disruption [11, 13–15]. It was also reported that Cer [AP] or saturated free fatty acid species with cholesterol normalization could discriminate among AD lesional sites, non-lesional sites and control skin, and showed a significant correlation with TEWL [16]. However, variations in those indicators within the skin of healthy subjects remain unknown.

With regard to the skin of healthy subjects, the levels of Cer [NP] and Cer [NDS] in the SC were positively correlated with capacitance and were negatively correlated with TEWL in cheek skin [17]. In dry winter skin, the levels of Cer [NP] and Cer [NH] correlated not only with the conductance but also with the visual dryness grade [18]. However, correlations between the levels and ratios of Cer subclasses and skin properties in healthy skin remain unclear [19]. Additionally, in spite of reports of the altered composition of Cer subclasses in the SC between barrier-disrupted and healthy skin, little is known about the composition of Cer subclasses in living KCs, especially during differentiation. These findings suggest characteristic features of Cer subclasses in the SC, in which some Cer subclasses, such as Cer [NP], Cer [NH] and Cer [NDS], show a positive correlation but others, such as Cer [NS] and Cer [AS], show a negative correlation with functional parameters of the SC. Therefore, the use of ratios among Cer subclasses in the SC, not their levels, might be a more sensitive marker for skin properties and epidermal differentiation.

The aim of our investigation was to identify new potential markers for barrier-disrupted and healthy skin that could be used to evaluate skin properties and epidermal differentiation in the SC. To address this, candidate markers were selected from barrier-disrupted skin, which shows distinct features of Cer subclasses compared to healthy skin. Subsequently, we ascertained whether these candidate markers are applicable to the facial skin of healthy Japanese female subjects.

## Methods

### Ethics

The protocol for patients with AD or Pso was approved by the Ethical Committees of the Kao Corporation and the Dokkyo Medical University as described in our previous report [8, 9]. Informed consent to participate in the study was obtained from each patient. The study protocol for Japanese healthy volunteers was approved by the Ethical Committee of the Kao Corporation. Signed informed consent was obtained from each subject after the procedures had been explained with documentation. These studies were conducted according to the Declaration of Helsinki Principles.

### Subjects

Cer profiles of patients with AD or Pso and age-matched healthy control subjects who had no history of skin disorders were obtained from our previous reports [8, 9]. Briefly, Cer profiles of AD patients (16–37 years old; average 28 years old; *n* = 8), healthy control subjects of the AD study (25–37 years old; average 31 years old; *n* = 7), Pso patients (36–74 years old; average 55 years old; *n* = 10), and healthy control subjects of the Pso study (39–76 years old, average 58 years old; *n* = 9) were examined. Additionally, healthy Japanese female volunteers (20–74 years old, average 46 years old; *n* = 210) without any skin disease diagnosis were enrolled as the test subjects.

### Cer and SC functional parameters analyses of barrier-disrupted skin

Analyses of Cer and SC functional parameters (TEWL and capacitance) of lesional and non-lesional skin of AD patients and their healthy control subjects were obtained from our previously reported data [8] as well as from Pso patients and their healthy control subjects [9]. The Cer subclasses were evaluated by tape-stripping of the SC as described elsewhere [6, 20]. The ratio of each Cer subclass (ng/μg protein) to Cer subclass (ng/μg protein) in SC obtained by tape-stripping was calculated.

### Skin measurements of healthy female facial skin

All measurements were conducted in an air-conditioned room (temperature 20 ± 4 °C; RH 40 ± 10%). After acclimatization for at least 35 min, TEWL and capacitance were measured with a Tewameter TM-210 and a Corneometer CM 825 (Courage and Khazaka Electronic, Koln, Germany), respectively. The spectral reflectance at the cheek was measured by the L*a*b* color system using a spectrophotometer CM-2002 (Konica-Minolta Inc., Tokyo, Japan). Digital images of the skin surface were obtained using a video microscope i-SCOPE USB2.0 with a 50x PL lens (Moritex International Co. Ltd., Tokyo, Japan). Skin texture and scaling were evaluated with a scoring method using digital photos. Skin texture (roughness/smoothness) was scored according to a range from 1.0 (rough) to 4.0 (smooth), including intermediate grades, for a total of 7 grades. Scaling was evaluated with 4 grades and was scored as: 0 (none), 1 (slight scaling), 2 (moderate scaling) and 3 (severe scaling).

### Preparation and LC-MS analysis of SC samples from healthy female facial skin

SC specimens were collected from the cheek area of each subject, followed by the profiling of Cer using reverse phase-liquid chromatography-mass spectrometry (RP-LC-MS) as described previously [21]. Briefly, SC specimens were collected by the tape-stripping method using 25 mm × 20 mm square pieces of polypropylene tape (465#40; Teraoka Seisakusho; Tokyo, Japan) with 4 consecutive strippings at the same region. Lipids were extracted from one-half of each specimen by methanol and were dried, then re-dissolved in chloroform/methanol/2-propanol (2/9/9). Types of Cer were analyzed using an Agilent 1100 Series LC/MSD SL system (Agilent Technologies, Santa Clara, CA, USA). N-heptadecanoyl-D-erythro-sphingosine (Avanti Polar Lipids, Alabaster, AL, USA) was used as an internal standard. An Agilent 1100 Series LC/MSD SL system equipped with a Multi ion source, ChemStation software, an 1100-well plate autosampler (Agilent Technologies) and a L-column ODS (2.1 mm i.d. × 150 mm, Chemicals Evaluation and Research Institute, Tokyo, Japan) was used. Chromatographic separation of the lipids was achieved at a flow rate of 0.2 mL/min using a binary gradient solvent system of mobile phase A (methanol/ water (1:1, v/v) containing 5 mM acetic acid and 10 mM ammonium acetate) and mobile phase B (2-propanol containing 5 mM acetic acid and 10 mM ammonium acetate). The mobile phases were consecutively programmed as follows: an isocratic elution of A (80%) and B (20%) for 1 min; a linear gradient of A (80–40%) and B (20–60%) between 1 and 2 min; a linear gradient of A (40–0%) and B (60–100%) between 2 and 30 min; an isocratic elution of A (0%) and B (100%) for 5 min; and an isocratic elution of A (80%) and B (20%) from 35 to 45 min for column equilibrium. The sample injection volume was 20 μL. The column temperature was maintained at 40 °C. MS parameters were as follows: polarity, negative ion mode; flow of heated dry nitrogen gas, 4.0 L/min; nebulizer gas pressure, 60 psi; heater temperature of nitrogen gas, 350 °C; vaporizer temperature, 200 °C; capillary voltage, 4000 V; charging voltage, 2000 V; fragmenter voltage, 200 V. Each type of ceramide was detected by selected ion monitoring (SIM) as m/z [M + CH3COO]- (Supplemental Table 1). Soluble proteins were evaluated from the residues after lipid extraction with 0.1 M NaOH and 1% (w/v) SDS (Wako Pure Chemical Industries, Ltd., Osaka, Japan) in aqueous solution at 60 °C for 2 h, then were subsequently neutralized with 2 M HCl (Wako Pure Chemical Industries). The quantity of protein was determined using a BCA kit (Thermoscientific, Rockford, IL, USA). The amount of each Cer subclass was adjusted per quantitated protein value. The ratio of each Cer subclass (ng/μg protein) to Cer subclass (ng/μg protein) in SC obtained by tape-stripping was calculated.

### Lipid analysis of human SC and living KCs

Normal skin samples were obtained from plastic surgery (*n* = 3, the back of a Japanese female aged 65, the thigh of a Japanese male aged 78, and an unknown site of a Japanese male aged 52), and the epidermis was separated from the dermis by heating at 60 °C for 2 min. The epidermis was then treated with 0.5% trypsin in PBS at 37 °C for 30 min to obtain the SC film and epidermal living KCs. Lipids in the SC and KCs were extracted by the Bligh-Dyer method [22]. Types of Cer were analysed as described previously [20] using an Agilent 1100 Series LC/MSD SL (single quadrupole) system. The ratio of the level of Cer [NP] (ng/μg protein) to Cer [NS] (ng/μg protein) and the protein level of each sample were analyzed as described above.

### Human epidermal keratinization culture model

The reconstructed human epidermal keratinization model (LabCyte EPI-Kit, Japan Tissue Engineering Co., Ltd. Aichi, Japan) was used according to the manufacturer’s instructions. Briefly, foreskin-derived human neonatal KCs were seeded in 24-well culture inserts (Corning Co., Ltd., Corning, NY, USA) with culture media provided by the manufacturer (assay medium; Japan Tissue Engineering Co., Ltd.) at 37 °C in a 5% CO_2_ atmosphere. Twenty-four h later, the medium was removed and the KCs were air-lifted (Day 1). After the air-lift, the KCs were cultured using assay medium supplemented with 25 μg/ml vitamin C until day 7. The medium was exchanged every other day and KCs were collected at 1, 2, 5 and 7 day(s) of culture.

### Lipid analysis of human epidermal keratinization culture model

Lipids were extracted from each KC sample by the Bligh-Dyer method [22]. Types of Cer were analyzed using RP-LC-MS as described above. Briefly, KC samples were extracted and dried, then re-dissolved in chloroform/methanol/2-propanol (2/9/9). Types of Cer were analyzed using an Agilent 1100 Series LC/MSD SL system (Agilent Technologies, Santa Clara, CA, USA). The Cer [NP]/[NS] ratio in each sample was analyzed as described above.

### Statistical analysis

For comparisons between skin properties and Cer amounts and/or ratios, Spearman’s correlation analysis was used. The Bonferroni correction was used for correction of multiple comparisons, giving a *p*-value < 0.05 as the level of statistical significance. Statistical significance between human SC and KCs was calculated by Student’s t-test. Statistical significance of KCs among culture periods was assessed using the Dunnett’s test. Data were analyzed using SPSS version X (SPSS, Chicago, IL, USA).

## Results

### The ratios of Cer subclasses detect differences among lesional, non-lesional and healthy control skin

**Fig. 1:**
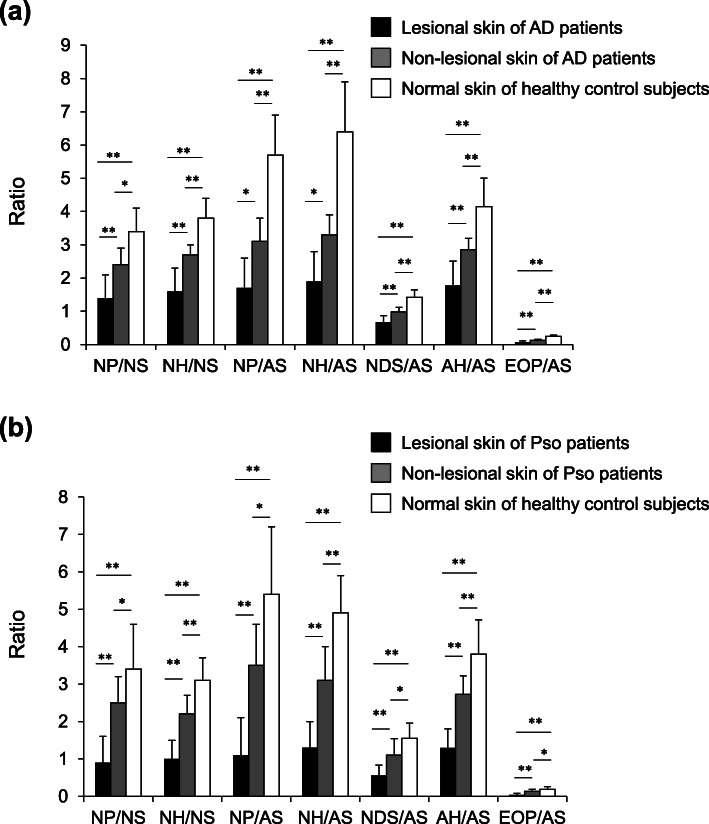
The ratios of Cer subclasses in the SC of patients with AD or Pso. The ratios of Cer subclasses in lesional skin of patients with AD (*n* = 8, black bars), in non-lesional skin of patients with AD (*n* = 8, gray bars) and in normal skin of healthy control subjects (*n* = 7, open bars). The ratios of Cer subclasses in lesional skin of patients with Pso (*n* = 10, black bars), in non-lesional skin of patients with Pso (*n* = 10, gray bars) and in normal skin of healthy control subjects (*n* = 9, open bars). All bars show means ± SD. Statistical significance determined by Bonferroni correction (*p < 0.05; **p < 0.01.) is marked

**Fig. 2:**
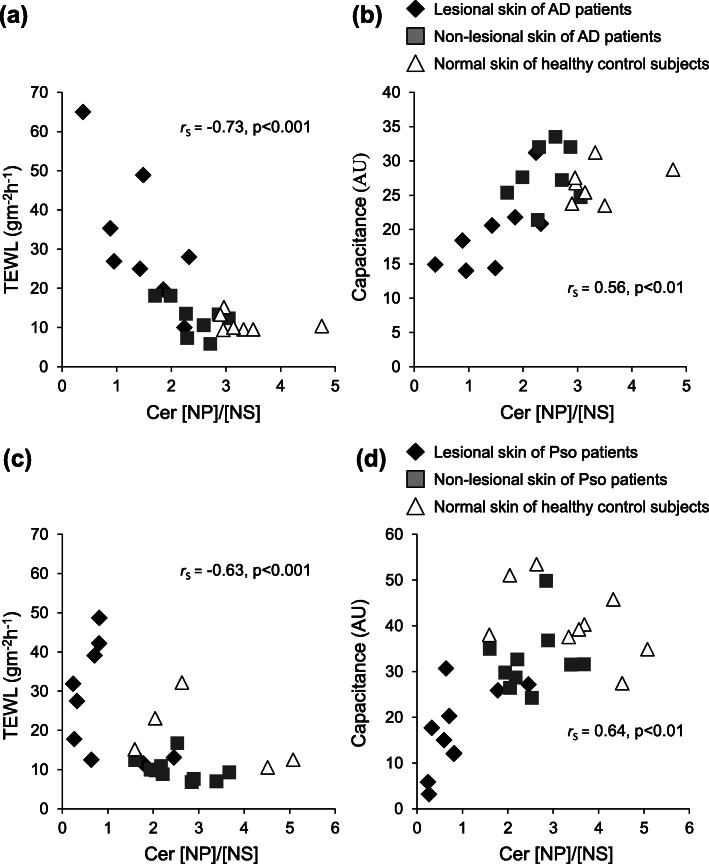
Correlations between the Cer [NP]/[NS] ratio and SC functional parameters of patients with AD or Pso. Correlations between the Cer [NP]/[NS] ratio with TEWL (*r*_S_ = − 0.73, *p* < 0.001) (**a**) and with capacitance (*r*_S_ = 0.56, *p* < 0.01) (**b**) in patients with AD and in healthy control subjects. Correlations between the Cer [NP]/[NS] ratio with TEWL (*r*_S_= − 0.63, *p* < 0.001) (**c**) and with capacitance value (*r*_S_ = 0.64, *p* < 0.01) (**d**) in patients with Pso and in healthy control subjects. AU, arbitrary units

We evaluated the ratio of each Cer subclass to the Cer subclass in the SC among normal skin of healthy control subjects, and non-lesional skin and lesional skin of patients with AD or Pso. The ratios of Cer [NP]/[NS], Cer [NH]/[NS], Cer [NP]/[AS], Cer [NH]/[AS], Cer [NDS]/[AS], Cer [AH]/[AS] and Cer [EOP]/[AS] were significantly different between non-lesional skin of AD patients and healthy control subjects as well as Pso patients (Fig. 1). However, the individual amounts of those Cer subclasses and the other ratios of Cer subclasses were not significantly different. In addition, these ratios of Cer subclasses showed statistically significant correlations with TEWL and capacitance values within the group of AD patients and its control subjects and the group of Pso patients and its control subjects (Table 1). The ratio of all phytosphingosine Cer (Cer [NP], Cer [AP] and Cer [EOP]) combined to all sphingosine Cer (Cer [NS], Cer [AS] and Cer [EOS]) also showed significant but lower correlations with TEWL in patients with AD (*r*_S_ = − 0.62, *p* < 0.001) or Pso (*r*_S_= − 0.60, *p* < 0.001) and in the healthy control subjects. Individual plots of the Cer [NP]/[NS] ratio versus the TEWL and capacitance values of AD or Pso patients are shown in Fig. 2, as detailed examples. The plots of non-lesional skin of patients were distributed almost intermediately between lesional skin of patients and normal skin of healthy control subjects.

**Table 1:** Correlation coefficients between SC functional parameters (TEWL and capacitance) and the ratio of Cer subclasses

Correlation coefficients (*r*_S_)					
		AD and control		Pso and control	
		TEWL (gm^− 2^ h^− 1^)	Capacitance (AU)	TEWL (gm^− 2^ h^− 1^)	Capacitance (AU)
Ratio	NP/NS	− 0.73***	0.56**	−0.63**	0.64***
	NH/NS	−0.78***	0.61**	−0.58**	0.73***
	NP/AS	−0.72***	0.57**	−0.63**	0.70***
	NH/AS	−0.76***	0.57**	−0.56**	0.75***
	NDS/AS	−0.63***	0.58**	−0.56**	0.70***
	AH/AS	−0.79***	0.64**	−0.62**	0.78***
	EOP/AS	−0.74***	0.62**	−0.69***	0.70***

The group of atopic dermatitis (AD) patients (*n* = 8) and healthy control subjects (*n* = 7). The group of psoriasis (Pso) patients (*n* = 10) and healthy control subjects (*n* = 9). ** and *** denote significant correlations of *p* < 0.01 and *p* < 0.001, respectively. Spearman’s correlation; the correlation coefficients (*r*_S_) and *p*-values are shown. *TEWL* Transepidermal water loss, *AU* Arbitrary units

### Cer [NP]/[NS] correlates with various skin biophysical parameters in healthy skin

**Fig. 3:**
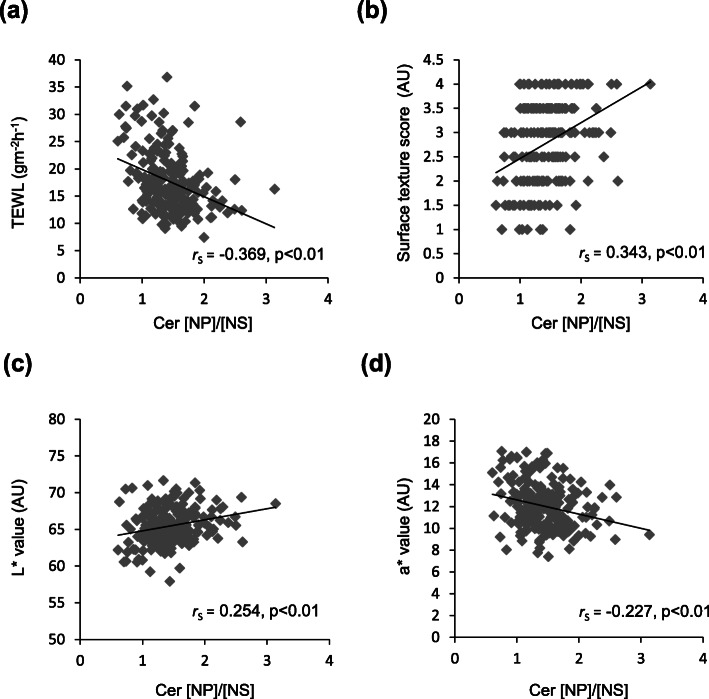
Correlations between the Cer [NP]/[NS] ratio and various skin properties in the healthy female facial skin. Correlation between the Cer [NP]/[NS] ratio and the SC functional parameter, TEWL (*r*_S_= − 0.369, *p* < 0.01) (**a**). Correlation between the Cer [NP]/[NS] ratio and the skin surface roughness parameter, surface texture score (roughness and smoothness) (*r*_S_= 0.343, *p* < 0.01) (**b**). Correlation between the Cer [NP]/[NS] ratio and the skin photometric color parameters, L* value (lightness) (*r*_S_ = 0.254, *p* < 0.01) (**c**) and a* value (redness) (*r*_S_ = − 0.227, *p* < 0.01) (**d**). Spearman’s correlation; the correlation coefficients (*r*_S_) and *p-*values are shown. TEWL, transepidermal water loss; AU, arbitrary units

Taken together, the ratios of Cer subclasses suggested their relevance to skin properties including SC barrier function even in healthy skin. Thus, we evaluated potential correlations among various skin properties and the ratios of Cer subclasses, Cer [NP]/[NS], Cer [NH]/[NS], Cer [NP]/[AS], Cer [NH]/[AS], Cer [NDS]/[AS], Cer [AH]/[AS] and Cer [EOP]/[AS] as candidates, in healthy female facial skin. As summarized in Table 2, the level of Cer [NP] and the ratios of Cer [NP]/[NS], Cer [NH]/[NS], Cer [NP]/[AS], Cer [NH]/[AS], Cer [NDS]/[AS], Cer [AH]/[AS] and Cer [EOP]/[AS] were negatively correlated with the TEWL value. The capacitance value was positively correlated with the levels of total Cer, Cer [NP] and Cer [NS] and the ratios of Cer [NP]/[NS], Cer [NH]/[NS] and Cer [EOP]/[AS]. The skin surface texture (roughness and smoothness, a higher score means a smoother texture) was also positively correlated with the levels of total Cer and Cer [NP], and the ratios of Cer [NP]/[NS], Cer [NH]/[NS], Cer [NP]/[AS] and Cer [NH]/[AS]. Among these Cer levels and ratios, the Cer [NP]/[NS] ratio showed the highest significant positive correlation with the surface texture. The scaling (a lower score means less scaling) showed a weak but significant negative correlation with Cer [NP] and the ratio of Cer [NP]/[NS]. In terms of skin color parameters, the levels of total Cer and Cer [NP] had significant positive correlations with L*(lightness). The level of Cer [NS] had a positive and the ratio of Cer [EOP]/[AS] had a negative correlation with a*(redness), respectively. The ratios of Cer [NP]/[NS], Cer [NH]/[NS] and Cer [NP]/[AS] were significantly correlated with L* and a* (positive and negative, respectively). On the other hand, the levels of total Cer and Cer [NS] had a negative correlation with b*(yellowness). As a result, only the Cer [NP]/[NS] ratio had significant correlations with all parameters of SC function and skin appearance except b* in healthy skin, which suggested that a higher Cer [NP]/[NS] ratio indicates healthy skin. Additionally, the Cer [NP]/[NS] ratio did not show a significant correlation with age (Supplemental Figure 1). Individual plots of the Cer [NP]/[NS] ratio versus TEWL, surface texture, L* value and a* value are shown in Fig. 3 (panels a, b, c and d, respectively).

**Table 2:** Correlation coefficients between skin features of Japanese healthy females and the ratio of Cer subclasses

Correlation coefficients (*r*_S_)								
		TEWL (gm^−2^ h^−1^)	Capacitance (AU)	Surface Texture (AU)	Scaling (AU)	L* (AU)	a* (AU)	b* (AU)
Level (ng/μg protein)	Total Cer	−0.087	0.284**	0.166*	−0.135	0.166*	0.015	−0.232**
	NP	−0.199**	0.289**	0.286**	−0.188**	0.270**	−0.135	−0.123
	NS	0.199**	0.181**	−0.004	−0.073	0.033	0.148*	−0.206**
Ratio (AU)	NP/NS	−0.369**	0.156*	0.343**	−0.167*	0.254**	−0.227**	0.018
	NH/NS	−0.445**	0.162*	0.238**	−0.121	0.148*	−0.142*	0.023
	NP/AS	−0.330**	0.121	0.269**	−0.111	0.181**	−0.198**	0.074
	NH/AS	−0.384**	0.129	0.192**	−0.085	0.076	−0.115	0.107
	NDS/AS	−0.275**	0.030	0.024	0.102	−0.087	−0.028	0.119
	AH/AS	−0.482**	0.057	0.056	0.114	−0.010	−0.100	0.132
	EOP/AS	−0.241**	0.201**	−0.166*	0.199**	0.122	−0.153*	0.011

Correlation coefficients between SC functional parameters (TEWL and capacitance), skin surface texture, scaling, color parameters (L*(lightness), a*(redness), b*((yellowness)) and the levels or ratios of Cer subclasses in the facial skin of healthy Japanese female subjects were evaluated. * and ** denote significant correlations of *p* < 0.05 and *p* < 0.01, respectively. Spearman’s correlation, the correlation coefficients (*r*_S_) and *p*-values are shown. TEWL, transepidermal water loss; AU, arbitrary units

### Cer [NP] and Cer [NP]/[NS] are higher in the SC than in KCs and increases during KC differentiation

**Fig. 4:**
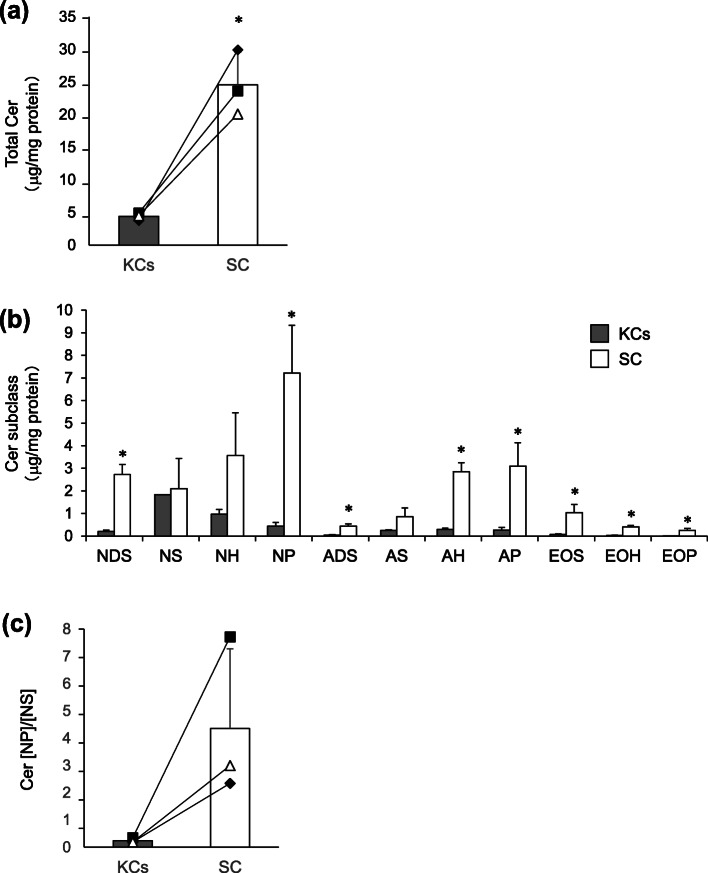
Levels of Cer subclasses and the Cer [NP]/[NS] ratio in the SC and in KCs. Levels of (**a**) total Cer, (**b**) each Cer subclass, and (**c**) the Cer [NP]/[NS] ratio in the SC and in KCs from normal human skin. Open and black bars indicate SC and KCs, respectively. ■ represents the back skin from a 65-year old female. ◆ represents the thigh skin from a 78-year old male. △ represents an unknown skin site from a 52-year old male. All bars show means ± SD. *N* = 3. Statistical significance determined by Student’s t-test (* *p* < 0.05)

**Fig. 5:**
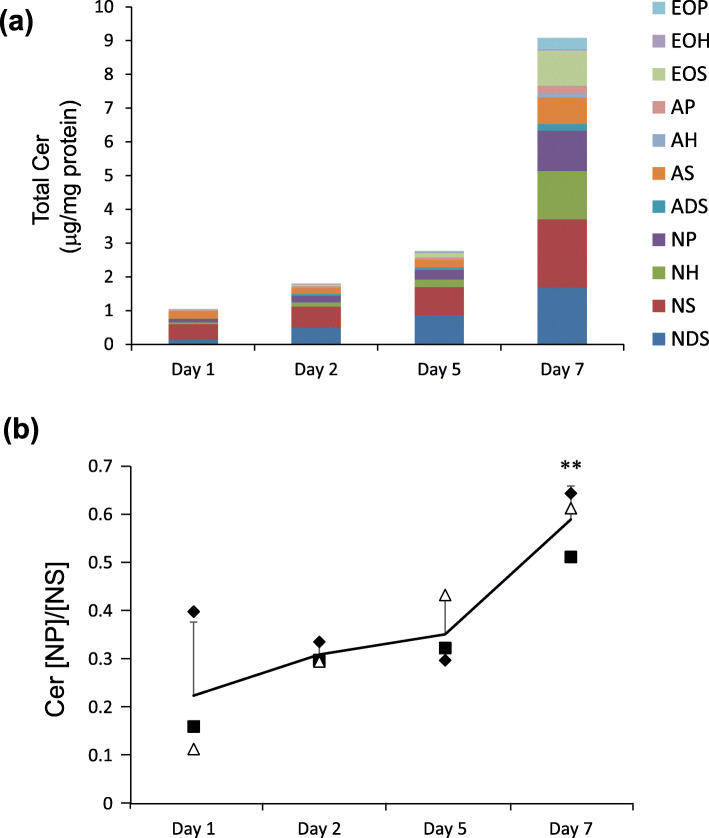
Cer subclass composition and the Cer [NP]/[NS] ratio during the differentiation of KCs. The level of total Cer and the composition of Cer subclasses (**a**), and the Cer [NP]/[NS] ratio (**b**) in the epidermal keratinization culture model. Day 1 indicates the date of the submerged culture. Cultures were raised to the air after day 1 to make more differentiated growth conditions for KCs. All bars show means ± SD, *N* = 3. Statistical significance determined by Dunnett’s test (** *p* < 0.01 vs. Day 1)

Disturbances in SC barrier function can be caused by changes in epidermal differentiation [23–25]. The significant correlation between the Cer [NP]/[NS] ratio and SC functional parameters suggested that the composition of Cer subclasses is also related to epidermal differentiation. To clarify whether the composition of Cer subclasses changes depending on the epidermal differentiation state, the detailed composition of Cer subclasses in human epidermis and in the reconstructed epidermal keratinization model was investigated. Compared to the SC and living KCs obtained from same subjects’ epidermis, the level of total Cer was significantly higher in the SC than in KCs (Fig. 4a). Further, among Cer subclasses, the levels of Cer [NDS], Cer [NP], Cer [ADS], Cer [AH], Cer [AP], Cer [EOS], Cer [EOH] and Cer [EOP], especially Cer [NP] were significantly higher in the SC than in KCs, while Cer [NS] was almost the same level in the SC and in KCs (Fig. 4b). Additionally, the Cer [NP]/[NS] ratio in the SC was approximately 18-times higher than in KCs (Fig. 4c). These results suggested that the state of cornification was reflected in the specific Cer subclass levels or ratios, such as Cer [NP] and/or Cer [NP]/[NS]. Thus, to investigate the alteration of Cer [NP] levels and the Cer [NP]/[NS] ratio during cornification, the reconstructed epidermal keratinization model was used. In the keratinization model, the total amount of Cer was increased accompanied by the diversification of Cer subclasses including Cer [NP] and Cer [NH] throughout the 7 day culture period (Fig. 5a), with Cer [NS] consistently being the major Cer subclass. Additionally, the Cer [NP]/[NS] ratio also increased over the 7 days of culture, and was significantly higher at day 7 (Fig. 5b).

## Discussion

The Cer composition of intercellular lipids in the SC is important for the skin barrier function. Although an altered Cer composition in the SC has been reported previously in some inflammatory skin diseases [8, 9, 11, 15, 26] and in a human skin reconstructed model [27], such as an increased Cer [AS] level and a decreased Cer [NP] level, little is known about the possible correlations between Cer subclass ratios and skin properties in barrier-disrupted skin and in healthy skin. As for the difference of Cer subclass in AD patients, a single Cer subclass, Cer [AP], normalized by cholesterol content had been reported to discriminate normal and non-lesional skin of Korean patients with AD [16]. As we show in this report, it is noteworthy that the Cer [NP]/[NS] ratio not only discriminates normal and non-lesional skin of patients with AD, but also shows a significant correlation with SC functional parameters both in barrier-disrupted skin and in healthy skin, which suggests that the Cer [NP]/[NS] ratio could be a potential marker to evaluate skin conditions even in healthy skin.

Our previous studies had demonstrated a significant correlation between the level of Cer [NP] and the relationship of skin surface roughness and texture with dry skin symptoms [18]. We report here for the first time that the Cer [NP]/[NS] ratio is related to the appearance of Japanese female facial skin. Only the Cer [NP]/[NS] ratio was significantly correlated with surface roughness parameters (surface texture and scaling) and skin photometric color parameters (L*(lightness) and a*(redness), but not b*(yellowness)) in healthy facial skin. Skin appearance is influenced by reflectance and light scattering at the surface, and hence the microstructure of the outer SC is important [28]. It is possible that improving the structural disorganization of the SC alters the surface reflectance and/or scattering of light, which affects the L* value [29, 30]. Interestingly, it is likely that the negative correlation between a* and the Cer [NP]/[NS] ratio suggests there is a relationship between inflammation and the composition of Cer subclasses, because the a* value partly reflects the skin condition of redness/erythema. It has also been reported that slight immune abnormalities induce skin redness/erythema accompanied by alterations of Cer subclass composition in the SC [31–34]. Recent studies have demonstrated that Th2 and Th1 cytokines are capable of interfering with the lipid organization via altered levels of enzymes involved in Cer synthesis (e.g. serine palmitoyl transferase-2, β-glucocerebrosidase and acid sphingomyelinase), thereby altering the Cer composition, such as the decrease in Cer [NP] and the increase in Cer [NS/NDS] [32–34]. We speculate that inflammation elevates a* and decreases Cer [NP]/[NS] via a perturbation of normal epidermal differentiation. Although further investigation is required to determine which factors contribute to skin appearance, it is interesting that a significant correlation between skin appearance and the characteristic Cer subclass ratio was found.

The results of this study demonstrated that the level of Cer [NP] and the Cer [NP]/[NS] ratio are higher in the SC compared with KCs in normal human skin and that Cer [NP] is the most abundant Cer subclass in the SC, while Cer [NS] is the most abundant Cer subclass in KCs. Although only three Japanese skin samples from different anatomic sites were analyzed, these results support the possibility that the Cer [NP]/[NS] ratio indicates the epidermal differentiation process, because it compared the SC with KCs from the same subjects. Previous studies also supported that Cer [NP] is the major Cer subclass in human SC from the forearm, leg and face [35, 36]. Moreover, the results also demonstrated that the Cer [NP]/[NS] ratio increases during the differentiation of KCs.

Regarding the mechanism(s) underlying that increase, sphingolipid C4 hydroxylase (DEGS2) is a key regulatory enzyme. In mammals, DEGS2 is involved in the biosynthesis of Cer [NP] though the C4-hydroxylation of Cer [NDS] [37], and it was reported that the expression of DEGS2 tightly regulates Cer [NP] production in differentiated KCs [38, 39]. Our results are in accord with those observations. On the other hand, the correlation of the ratio of all phytosphingosine Cer (Cer [NP], Cer [AP] and Cer [EOP]) combined to all sphingosine Cer (Cer [NS], Cer [AS] and Cer [EOS]) and TEWL with AD showed a significant but not a stronger correlation coefficient compared with other Cer subclass ratios as shown in Table 1. It remains unclear whether DEGS2 is directly involved in the synthesis of [A] and/or [EO] type phytoceramides. Moreover, in addition to the de novo synthesis pathway, the levels of Cer subclasses in the SC could also be regulated by ceramidases [40, 41]. Therefore, it would be possible that the ratio of Cer [NP]/[NS] showed a better correlation with SC functional parameters compared to the ratio of all phytosphingosine Cer combined to all sphingosine Cer.

This is an initial report with limited skin samples, and further investigation will be necessary to prove that the Cer [NP]/[NS] ratio is an appropriate marker for skin properties and epidermal differentiation, by examining more skin specimens, locations and so on. However, we found no significant correlation between age and the Cer [NP]/[NS] ratio in healthy Japanese female facial skin (Supplemental Figure 1). Thus, the Cer [NP]/[NS] ratio is likely to be less affected at least with regard to age. Additionally, further investigation is needed to clarify whether the Cer [NP]/[NS] ratio is a predictive marker such as a long-term observation study of AD patients, and to confirm whether the Cer [NP]/[NS] ratio also correlates with SC function and skin appearance even in healthy facial skin, such as in other population groups and in males.

## Conclusions

The Cer [NP]/[NS] ratio in the SC is suggested as a potential marker related to skin properties and epidermal differentiation, with a higher Cer [NP]/[NS] ratio indicating healthy skin conditions and a lower Cer [NP]/[NS] ratio indicating poor or undifferentiated skin conditions in barrier-disrupted skin as well as in apparently normal skin. Thus, the Cer [NP]/[NS] ratio is a potential marker for the prevention of barrier impairment and/or the improvement of healthy skin conditions.

## Supplementary information

**Additional file 1: Supplemental Table 1.xls.** Selected m/z values for ceramide species in the MS analysis of SC samples from healthy female facial skin.

**Additional file 2: Supplemental Figure 1.pptx.** Correlations between the Cer [NP]/[NS] ratio and age in healthy female facial skin.

## Data Availability

The datasets used and/or analysed during the current study are available from the corresponding author on reasonable request.

## Abbreviations

SC: Stratum corneum
Cer: Ceramide
KC: Keratinocytes
AD: Atopic dermatitis
Pso: Psoriasis
