# Supplementary figures and images for: The ceramide [NP]/[NS] ratio in the stratum corneum is a potential marker for skin properties and epidermal differentiation
Source: BMC Dermatol. 2020 Aug 31;20:6. doi: 10.1186/s12895-020-00102-1 (PMC7461267; doi:10.1186/s12895-020-00102-1)

## Slide 1
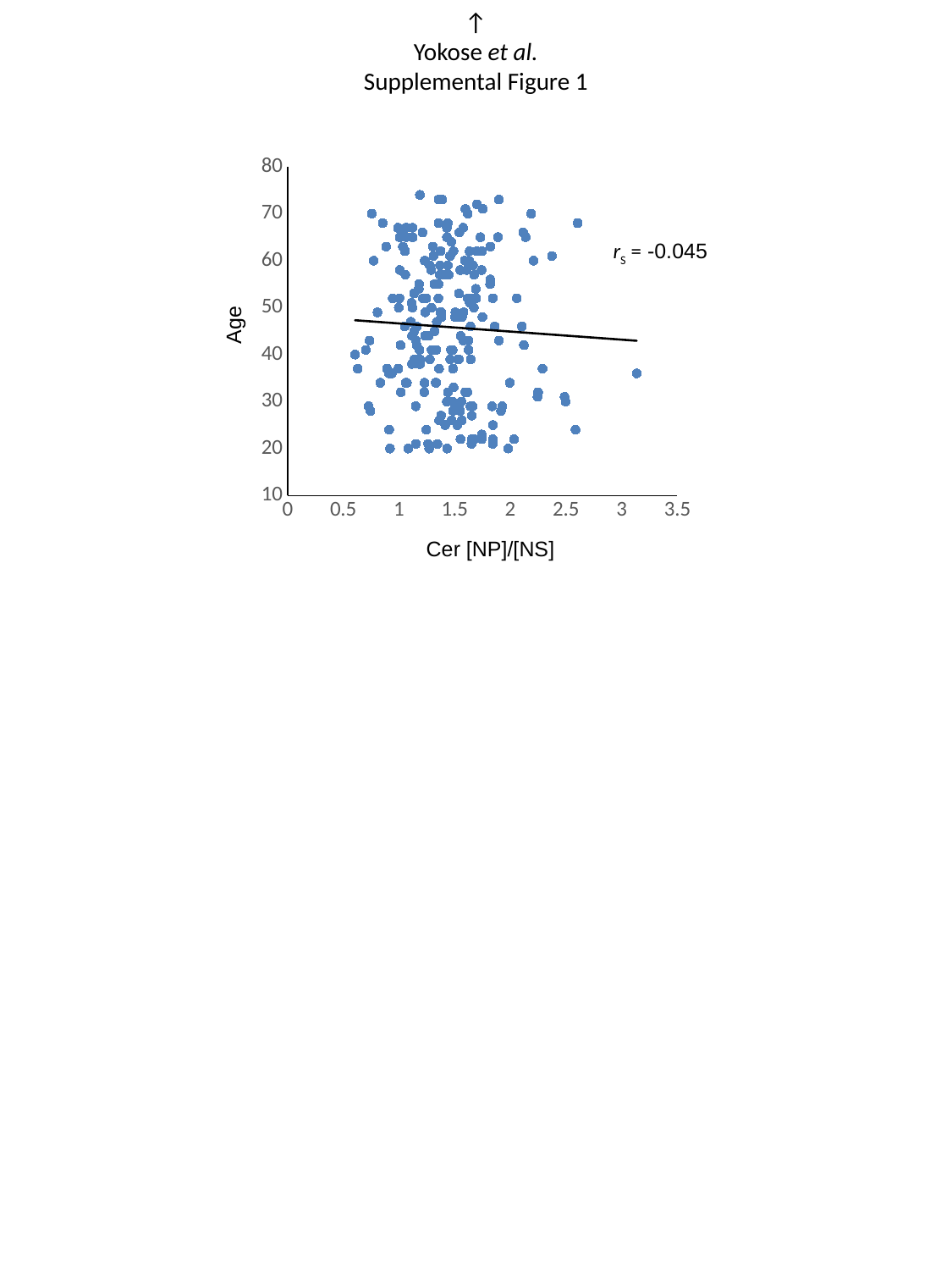

↑
Yokose et al.
Supplemental Figure 1
### Chart
| Category | 年齢 |
|---|---|rS = -0.045
Age
Cer [NP]/[NS]

Supplement: Supplementary file 2 — Additional file 2: Supplemental Figure 1.pptx. Correlations between the Cer [NP]/[NS] ratio and age in healthy female facial skin. [file 12895_2020_102_MOESM2_ESM.pptx]
